# Climate change, urbanisation and transmission potential: *Aedes aegypti* mosquito projections forecast future arboviral disease hotspots in Brazil

**DOI:** 10.1371/journal.pntd.0013415

**Published:** 2025-09-18

**Authors:** Katherine Heath, Lincoln Muniz Alves, Michael B. Bonsall

**Affiliations:** 1 Burnet Institute, Melbourne, Victoria, Australia; 2 Mathematical Ecology Research Group, Department of Biology, University of Oxford, Oxford, United Kingdom; 3 Centro de Ciência do Sistema Terrestre CCST, Instituto Nacional de Pesquisas Espaciais INPE, São José dos Campos, Brazil; 4 St. Peter’s College, Oxford, United Kingdom; Egerton University, KENYA

## Abstract

**Background:**

Climate change and urban expansion pose significant challenges to controlling *Aedes aegypti* mosquito populations, a primary vector of arboviruses such as dengue, Zika, and chikungunya. This study aims assess how climate and anthropogenic factors will jointly shape *Ae. aegypti* densities in Brazil, which is crucial to forecasting transmission risks and informing public health strategies.

**Methods:**

This study combined a biologically informed, stage-structured delay-differential equation model with climate and anthropogenic data. Climate projections from the Coupled Model Intercomparison Project Phase 6 under different Shared Socioeconomic Pathways (SSPs) were used to forecast future climate scenarios from 2024 to 2080. Boosted Regression Trees integrated anthropogenic factors like urbanisation, population growth, and urban accessibility. Model outputs were validated with entomological surveillance data, and the basic reproductive number for dengue fever was used to assess changes in disease transmission potential.

**Findings:**

Our findings predicted that *Ae. aegypti* mosquito density will increase nationally, but unevenly, exceeding thermal limits in North Brazil while rising substantially in the South and Southeast. Increases in density were particularly pronounced under high greenhouse gas emission scenario SSP5-8.5 (up to 92% in the Southeast). These trends were projected to elevate the transmission potential for dengue fever, with Southeast Brazil facing the biggest increases due to mosquito population growth outpacing human population expansion. Validation against historical data confirmed model robustness.

**Interpretation:**

By directly linking mosquito abundance to SSP-specific emissions trajectories, our results show that climate mitigation can markedly reduce disease risk. Shifting from SSP5-8.5 to SSP1-2.6 could cut projected mosquito density increases from 31% to 11% nationally by 2080. The model’s spatial granularity and integration of local administrative boundaries support its utility for national and sub-national health planning. Addressing compounded risks in vulnerable peri-urban and rural populations will require coordinated interventions that span climate policy, vector control, and health equity.

## Author summary

*Aedes aegypti* mosquitoes transmit viruses like dengue, Zika, and chikungunya, which are increasing in many parts of the world, including Brazil. Climate change and rapid urban growth are likely to increase the risk of mosquito-borne disease outbreaks in the future. In this study, we developed a model that combines mosquito biology with climate and urbanisation data to estimate how mosquito populations might change in Brazil under different future climate and development scenarios. Our model predicts that mosquito populations will increase significantly, especially in the South and Southeast of Brazil, where conditions are becoming more favourable for mosquito survival and reproduction. We also found that the risk of disease transmission is expected to rise as mosquito numbers grow faster than the human population. These findings suggest that both climate change and human development patterns are likely to increase the burden of mosquito-borne diseases. By showing where and when mosquito risks are likely to be greatest, our study can help public health authorities prepare and respond more effectively. It also highlights the importance of climate action to reduce future health risks.

## Introduction

The mosquito *Aedes aegypti*, a primary vector of arboviruses such as dengue, Zika and chikungunya, poses a growing public health challenge in the context of climate change [1]. Understanding the mechanisms that drive mosquito population dynamics is vital for developing targeted and effective interventions to prevent arboviral diseases and mitigate their public health impact.

Globally, 2024 had the largest number of dengue cases on record [2]. Brazil, which has the world’s largest burdens of both Zika and dengue, is particularly vulnerable to this disease due to its climatic suitability for *Ae. aegypti* and rapid urban growth [3]. While *Ae. albopictus* plays a growing role in parts of Europe and Asia, *Ae. aegypti* remains the dominant vector in tropical and urban regions such as Brazil*. Ae. aegypti* is highly anthropophilic and thrives in densely populated urban environments, where artificial containers provide abundant breeding sites and close human contact facilitates transmission.

Temperature and precipitation directly influence *Ae. aegypti* populations through effects on development, survival, and reproductive rates [4,5]. Whilst elevated temperatures generally accelerate *Ae. aegypti* development, extreme heat can have detrimental effects [4]. Recent evidence has shown that climate-driven shifts in mosquito-borne disease transmission are already occurring in Brazil, with rising temperatures and altered precipitation patterns contributing to expansion of dengue [6].

Several models have projected future climatic suitability for *Ae. aegypti* or have estimated arboviral disease risk using climatic data, offering powerful tools for large-scale projections [7–11]. However, the relationship between climate and mosquito life history is complex, non-linear, and difficult to capture without mechanistic, biologically informed modelling approaches [10]. Further, climatic drivers are modulated by human activities, such as urban infrastructure and socioeconomic conditions, highlighting the need for interdisciplinary approaches [12–14]. While recent advances have incorporated anthropogenic factors, gaps remain in mechanistically linking these factors with the non-linear effects of climatic variables on mosquito ecology at large scales [15,16].

This study aims to address critical gaps in understanding the interplay between climatic and anthropogenic factors driving *Ae. aegypti* population dynamics by developing a biologically informed, integrated modelling framework. To evaluate future changes, we leveraged the Shared Socioeconomic Pathways (SSPs), which represent alternative climate and societal scenarios characterised by differing greenhouse gas emission trajectories as well as socio-economic factors such as population growth, urbanisation, and climate change mitigation [17,18]. We focus on four SSPs reflecting a spectrum of futures: SSP1-2.6 (low emissions, strong mitigation), SSP2-4.5 and SSP3-7.0 (intermediate scenarios), and SSP5-8.5 (high emissions, limited mitigation). These scenarios vary in projected temperature increases, precipitation patterns, and socio-economic conditions, offering a comprehensive basis for projecting mosquito dynamics across Brazil. [17,18]

The primary objective is to assess how climate and anthropogenic factors will jointly shape *Ae. aegypti* densities in Brazil. The secondary objective is to estimate associated changes in arboviral disease transmission potential.

## Materials and methods

### Main model

A stage-structured delay-differential equation (DDE) model was used to model adult and juvenile *Ae. aegypti* populations, which were governed by recruitment, maturation, and stage-specific mortality:


$$\frac{dJ(t)}{dt}=R(t)-M(t)-\left(\mu_{J}(t)+\delta (t)J(t)\right)J(t)$$ (1)

$$\frac{dA(t)}{dt}=M(t)-\mu_{A}(t)A(t)$$ (2)

Parameters R(t) and M(t) denote the time-dependent rates of recruitment (egg-laying) and maturation (pupation) between adult A(t), and juvenile J(t), stages at time, t. Parameters $\mu_{J}(t)$ and $\mu_{A}(t)$ represent the mortality rates, and δ(t) captures density-dependent mortality. Recruitment, R(t)*,* and maturation, M(t), were defined as:


$$R(t)=\;\frac{A(t)b(t)}{2}$$ (3)

$$M(t)=R(t-\tau_{J}(t))S_{J}(t)\frac{g_{J}(t)}{g_{J}(t-\tau_{J}(t))}$$ (4)

where b(t) represents the number of eggs laid per female at time, t. R(t) is scaled by a factor of ½ to account for the sex ratio of the population, thereby implicitly assuming that the female:male emergence ratio is 1:1. Parameter $S_{J}(t)$ represents the juvenile survival rate*.* The rate of change in the juvenile development time from hatch to emergence, $\tau_{J}(t)$*,* was defined as follows:


$$\frac{d\tau_{J}(t)}{dt}=1-\frac{g_{J}(t)}{g_{J}(t-\tau_{J}(t))}$$ (5)

where $g_{J}(t)$ is the juvenile development rate and $g_{J}(t)=\frac{\text{1}}{\tau_{J}(t)}$. The instantaneous juvenile survival rate was defined as follows.


$$S_{J}(t)=e^{-\int_{t-\tau_{J}(t)}^{t}\left(\delta (t)J(t)+\mu_{J}(t)\right)dt}$$ (6)

from which the rate of change in juvenile survival was defined as follows:


$$\frac{dS_{J}(t)}{dt}=S_{J}(t)\left[\left(\delta \left(t-\tau_{J}(t)\right)J\left(t-\tau_{J}(t)\right)+\mu_{J}\left(t-\tau_{J}(t)\right)\right)\frac{g_{J}(t)}{g_{J}(t-\tau_{J}(t))}-\delta (t)J(t)-\mu_{J}(t)\right]\;$$ (7)

A full derivation of the rate of change in survival is available in S1 Text. Each model iteration corresponded to 24 hours All analyses were conducted using R version 4.0 [19]. Details of specific packages and functions used is given in S2 Text.

### Temperature variation

Four parameters were temperature dependent: adult fecundity, b(t), adult mortality, $\mu_{A}(t)$, juvenile development rate, $g_{J}(t)$, and juvenile mortality, $\mu_{J}(t)$.

Mordecai et al. (2017) fitted thermal response models to empirical data for *Ae. aegypti* and *Aedes albopictus*, defining adult fecundity, b(t), using a Brière function [5]:


$$b(t)=c_{b}T(t)\left(T(t)-T_{0b}\right)\sqrt{\left(T_{Mb}-T(t)\right)}$$ (8)

where $T_{0b}$ and $T_{Mb}$ are the thermal limits, $c_{b}$ is a constant, and T(t) is temperature in degrees Celsius (°C).

Alternative functions were fitted for $\mu_{A}(t)$ and $g_{J}(t)$ using empirical data extracted from the literature across a spectrum of temperatures.

A Gaussian function modelled adult mortality:


$$\mu_{A}(t)=\frac{1}{ke^{-\frac{1}{2}{\left(\frac{T(t)-\gamma }{\sigma }\right)}^{2}}}$$ (9)

The juvenile development time, $\tau_{J}(t)$, followed a 2^nd^-degree polynomial:


$$\tau_{J}(t)=\beta_{0g}+\beta_{1g}T(t)+\beta_{2g}{T(t)}^{2}$$ (10)

facilitating estimation of the juvenile development rate, $g_{J}(t)=\frac{\text{1}}{\tau_{J}(t)}$.

A fourth-degree polynomial was used to describe temperature-dependent juvenile mortality:


$$\mu_{J}(t)=\beta_{0\mu }+\beta_{1\mu }T(t)+\beta_{2\mu }{T(t)}^{2}+\beta_{3\mu }{T(t)}^{3}+\beta_{4\mu }{T(t)}^{4}$$ (11)

Full derivations of Equations 9–11 are given in S3 Text, and parameters, confidence intervals, and references for extracted data are presented in S1 Table.

### Precipitation variation

Rainfall was assumed to enhance the environment’s carrying capacity by creating and maintaining oviposition sites. The carrying capacity, *K*, was modelled as proportional to exponentially weighted past daily rainfall (mm day^-1^ p(t), defined as:


$$\begin{array}{c} K(t)=\lambda \frac{1}{\omega \left(1-e^{\frac{-t}{\omega }}\right)}\int_{t-30}^{t}e^{\frac{-\left(t-t^{\prime }\right)}{\omega }}p\left(t^{\prime }\right)dt^{\prime } \end{array}$$ (12)

where ω represents the number of previous days contributing to the current carrying capacity and λ is a constant. Exponential weighting was applied because it has been shown to provide a better fit to entomological observations than linear weighting [20]. Density-dependent mortality was defined as $\delta (t)=\frac{1}{K(t)}$. Further details are given in S4 Text and parameter estimates are presented in S1 Table.

### Climate data

Monthly average temperature (°C) and precipitation (mm day^-1^) data were sourced from the Coupled Model Intercomparison Project Phase 6 (CMIP6). CMIP6 incorporates five SSPs and four Representative Concentration Pathways (RCPs). SSPs describe socioeconomic pathways, while RCPs define greenhouse gas emissions; combined, they create climate scenarios in CMIP6. Four ‘Tier 1’ scenarios, defined by The Scenario Model Intercomparison Project, range from low emissions with strong climate mitigation efforts (SSP1-2.6) to high emissions driven by rapid economic growth and reliance on fossil fuels (SSP5-8.5).

Under CMIP6 scenarios, Brazil is expected to experience increased temperatures across all SSPs, ranging from ~1.5°C under SSP1-2.6 to up to ~6 under SSP5-8.5 by the end of the century. Precipitation changes are more regionally and seasonally variable: low-emission scenarios show minor changes, while high-emission scenarios indicate greater variability including drier dry seasons in some areas and intense rainfall in others.

Daily temperature and precipitation estimates were derived from each monthly data point for the Tier 1 scenarios SSP1-2.6, SSP2-4.5, SSP3-7.0 and SSP5-8.5 using cubic spline interpolation.

### Anthropogenic variables

Since *Ae. aegypti* is highly anthropophilic, Boosted Regression Trees (BRTs) were used to isolate the effects of anthropogenic variables on its occurrence probability. The response variable was the median municipal building index (2018–2021), which represents the proportion of sampled buildings infested with *Ae. aegypti* during the 2018–2021 sampling period in each Brazilian municipality. These data were collected by the Brazilian Ministry of Health using the LIRAa protocol, for which a detailed description is given in S5 Text.

To train BRT models, several key covariates were used. Mean, minimum, and maximum annual temperatures for each Brazilian municipality were derived from CMIP6 data for 2024. Mean municipal urban accessibility, defined as the travel time (in minutes) to the nearest city with over 50,000 inhabitants, was extracted from the urban accessibility map by Weiss et al. (2018) [21]. Projected municipal population sizes for 2024–2050 were obtained from global population projections by Jones & O’Neill (2016) for the SSP1, SSP2, SSP3, and SSP5 scenarios, with SSP2 data for 2024 used for model training [22]. Projections of mean municipal urban land cover for 2024–2050 were taken from Zhou et al. (2019), with 2024 data used to train the BRTs [23].

BRT models were trained with ten-fold cross-validation and predictions were generated at a 5km² resolution. Temperature was fixed at 26 °C during BRT prediction to provide a biologically relevant baseline near the optimal range for *Ae. aegypti* development. This standardisation isolated the effects of anthropogenic variables by removing spatial variation in temperature, allowing clearer interpretation of human-driven influences. Further details on implementation of BRTs are given in the S6 Text, and a description of datasets used throughout the study is presented in S2 Table.

To avoid pseudo-replication, model-predicted *Ae. aegypti* abundance from the main model was converted into mosquito density (mosquitoes per km²), ensuring each observation reflected an independent, area-standardised measure of abundance. Density estimates were spatially smoothed using fixed rank kriging. The estimated building index for each grid cell was then multiplied by the kriged mosquito density estimates to incorporate anthropogenic variables into the final model outputs.

### Model validation against existing time series

To validate model predictions, a comprehensive literature search was conducted to identify existing *Ae. aegypti* surveillance data across Brazil. Data were extracted for nine locations: Campo Grande, Duque de Caxias, Governador Valadares, Manaus, Novo Iguaçu, Paranamarim, Porto Alegre, Sete Lagoas and Vitória [24–27]. These data were collected using either BG-Sentinal traps (BGS) (Biogents AG, Germany) or MosquiTraps (MQT) (Ecovec Ltda., Brazil).

Temporal trends in model-predicted *Ae. aegypti* density were compared against trap data using a modified Chelton method, which assesses the correlation between two time series, adjusting the threshold for significance via the degrees of freedom to deal with cases of strong autocorrelation [28]. Further details on the inclusion criteria for published surveillance data and the modified Chelton method employed are available in S7 Text.

### Calculation of *R*_*0*_

We used a simplified Ross-Macdonald model to demonstrate how our *Ae. aegypti* density predictions can be applied. The model was as follows:


$$\frac{dH(t)}{dt}=aBV(t)\frac{A(t)}{2N(t)}\left(1-H(t)\right)-rH(t)$$ (13)

$$\frac{dV(t)}{dt}=aCH(t)\left(1-V(t)\right)-\mu_{A}(t)V(t)$$ (14)

where H(t) and V(t) represent the infected proportion of human and vector populations respectively. a is the mosquito biting rate, B is the probability of a bite from an infectious mosquito infecting a human, C is the probability of an infectious blood meal infecting a mosquito and r is the human recovery rate. A(t) is the number of adult mosquitoes, N(t) is the human population size and $\mu_{A}(t)$ is the temperature-dependent adult mosquito mortality rate (see Equation 9). The model was parameterised based on Alphey et al. (2011), with values given in S3 Table [29].

By deriving the Jacobian matrix of the two-compartment model, the stability of the disease-free state is defined by the basic reproductive number, $R_{0}$:


$$R_{0}=Det[\begin{array}{cc} -r & \frac{aBA^{*}}{2N^{*}}\\ aC & -{\mu_{A}}^{*} \end{array}]$$ (15)

where $A^{*}$, $N^{*}$ and ${\mu_{A}}^{*}$ are the mosquito abundance, human population size, and adult mosquito mortality at time, *t*. Full derivation is given in the S8 Text. A positive $R_{0}$ indicates instability of the disease-free state, while a negative $R_{0}$ indicates stabili*t*y. Equation 15 was applied to each Brazilian municipality. Projections of future human population size, $N^{*}$, for SSP2 were extracted from Jones & O’Neill (2016) [22].

## Results

### Predicted spatiotemporal changes in mosquito density

**Fig 1:**
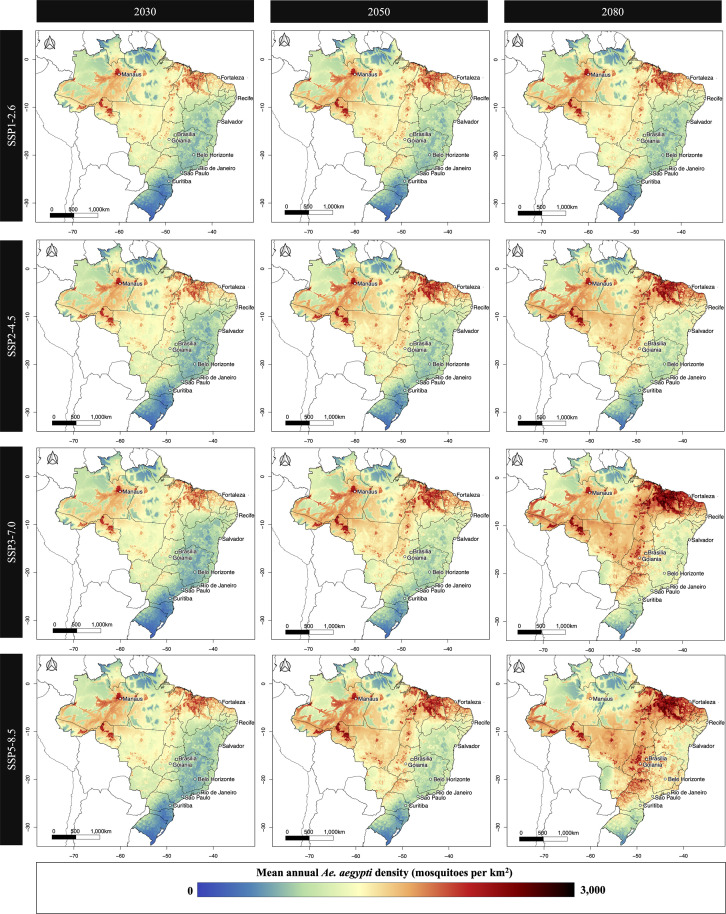
Mean annual model-estimated *Ae. aegypti* mosquito density (mosquitoes per km^2^) in Brazil. Results are presented for, from left to right, years 2030, 2050 and 2080. From top to bottom, figures present results for four future greenhouse gas emission scenarios: SSP1-2.6 (low emissions, strong mitigation), SSP2-4.5 and SSP3-7.0 (intermediate scenarios), and SSP5-8.5 (high emissions, limited mitigation). National and state borders are based on shapefiles from geoBoudaries (https://www.geoboundaries.org/), which are provided under the CC BY 4.0 license (https://creativecommons.org/licenses/by/4.0/) [37].

Fig 1 presents predicted mean annual *Ae. aegypti* density across Brazil for the years 2030, 2050 and 2080 under SSP1-2.6, SSP2-4.5, SSP3-7.0, and SSP5-8.5. Under all scenarios, high-density mosquito areas are consistently concentrated in the North and Central-west regions of Brazil, particularly in and around the Amazon basin and parts of the Northeast. The spatial extent and intensity of high-density zones was projected to increase markedly under higher-emissions scenarios SSP3-7.0 and SSP5-8.5.

By 2080, SSP3-7.0 and SSP5-8.5 projected expansion of high mosquito density into previously lower density regions in the Southeast and parts of the South, including areas surrounding major urban centres such as São Paulo, Belo Horizonte and Rio de Janeiro. In contrast, SSP1-2.6 showed relatively limited expansion of high-density zones. Model-estimated mean annual *Ae. aegypti* densities over time and by SSP are for major cities, regions and states are presented in S4–S6 Tables.

### National and subnational increases in mosquito density

Table 1 presents the projected fold-change in mosquito density relative to 2024 levels at the national level. Nationally, mosquito density was projected to increase progressively across all scenarios, although the magnitude of change varied markedly. Under the low-emission scenario (SSP1-2.6), national mosquito density rose modestly from 4% in 2030 to 11% in 2080. In contrast, high-emission scenarios SSP3-7.0 and SSP5-8.5 project sharper increases, increasing by 32% and 31%, respectively, by 2080.

**Table 1:** Model-estimated fold-change in mean annual *Ae. aegypti* density (mosquitoes per km^2^) in Brazil’s five geographic regions, and nationally, for 2030, 2050 and 2080 under four greenhouse gas emission scenarios: SSP1–2.6 (low), SSP2–4.5 and SSP3–7.0 (intermediate), and SSP5–8.5 (high).

SSP	Year	Region					
**North**	**Northeast**	**Southeast**	**Central-west**	**South**	**National**
**1-2.6**	**2030**	1.02	1.06	1.10	1.06	1.03	**1.04**
	**2050**	1.07	1.11	1.16	1.10	1.16	**1.10**
	**2080**	1.08	1.12	1.17	1.11	1.21	**1.11**
**2-4.5**	**2030**	1.03	1.05	1.07	1.04	1.08	**1.04**
	**2050**	1.09	1.17	1.21	1.14	1.24	**1.13**
	**2080**	1.13	1.32	1.37	1.21	1.43	**1.21**
**3-7.0**	**2030**	1.01	1.04	1.04	1.02	1.05	**1.02**
	**2050**	1.11	1.21	1.27	1.15	1.32	**1.16**
	**2080**	1.18	1.46	1.68	1.31	1.76	**1.32**
**5-8.5**	**2030**	1.04	1.05	1.03	1.03	1.04	**1.04**
	**2050**	1.13	1.25	1.33	1.2	1.33	**1.19**
	**2080**	1.09	1.51	1.92	1.32	1.89	**1.31**

**Fig 2:**
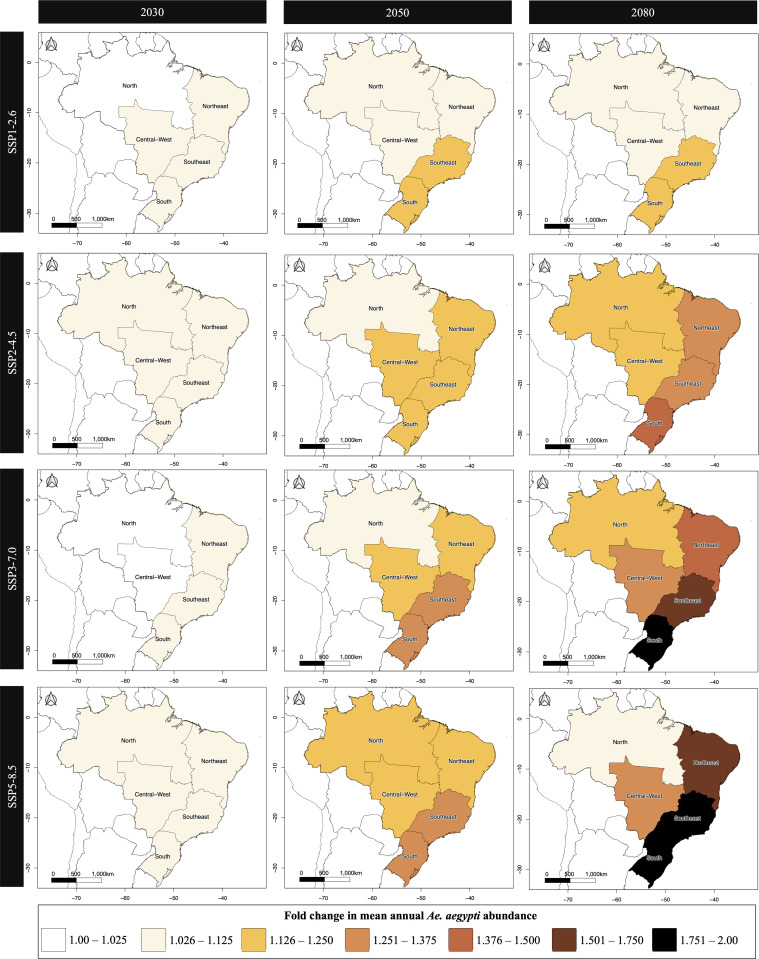
Fold change in mean annual model-estimated *Ae. aegypti* mosquito density (mosquitoes per km^2^) from 2024 by region of Brazil (North, Northeast, Central-West, Southeast and South). Results are presented for, from left to right, years 2030, 2050 and 2080. From top to bottom, figures present results for four future greenhouse gas emission scenarios: SSP1-2.6 (low emissions, strong mitigation), SSP2-4.5 and SSP3-7.0 (intermediate scenarios), and SSP5-8.5 (high emissions, limited mitigation). National and state borders are based on shapefiles from geoBoundaries (https://www.geoboundaries.org/), which are provided under the CC BY 4.0 license (https://creativecommons.org/licenses/by/4.0/) [37]. Regional boundaries were generated in QGIS (v3.36.2) by aggregating state-level polygons to create custom regional shapefiles [38].

Fig 2 presents the projected fold-change in mosquito density relative to 2024 levels across Brazil’s five geographic regions, the data for which are presented in Table 1. At the subnational level, mosquito density increases were projected to be modest by 2030 but grew substantially by 2050 and 2080, especially under high-emission scenarios. The South and Southeast showed the largest increases in *Ae. aegypti* density by 2080, rising by 89% and 92%, respectively, under SSP5-8.5. The Northeast and Central-west also exhibited strong growth in density over time, especially under SSP3-7.0 and SSP5-8.5, with increases exceeding 30% by 2080.

In contrast, the North region – which includes the major city of Manaus – showed more modest increases in *Ae. aegypti* density. In all scenarios except SSP5-8.5, the North region experienced upwards trends in mosquito density until 2080. Under SSP5-8.5, density increased by 13% by 2050 but rose only 9% by 2080 compared to 2024. This contrasts with 2080 increases of 18% under SSP3-7.0, 13% under SSP2-4.5, and 8% under SSP1-2.6. Model-estimated fold-changes in mean annual *Ae. aegypti* density from 2024 in major cities, regions and states are presented in S7–S9 Tables.

### New seasonal patterns of *Ae. aegypti*

**Fig 3:**
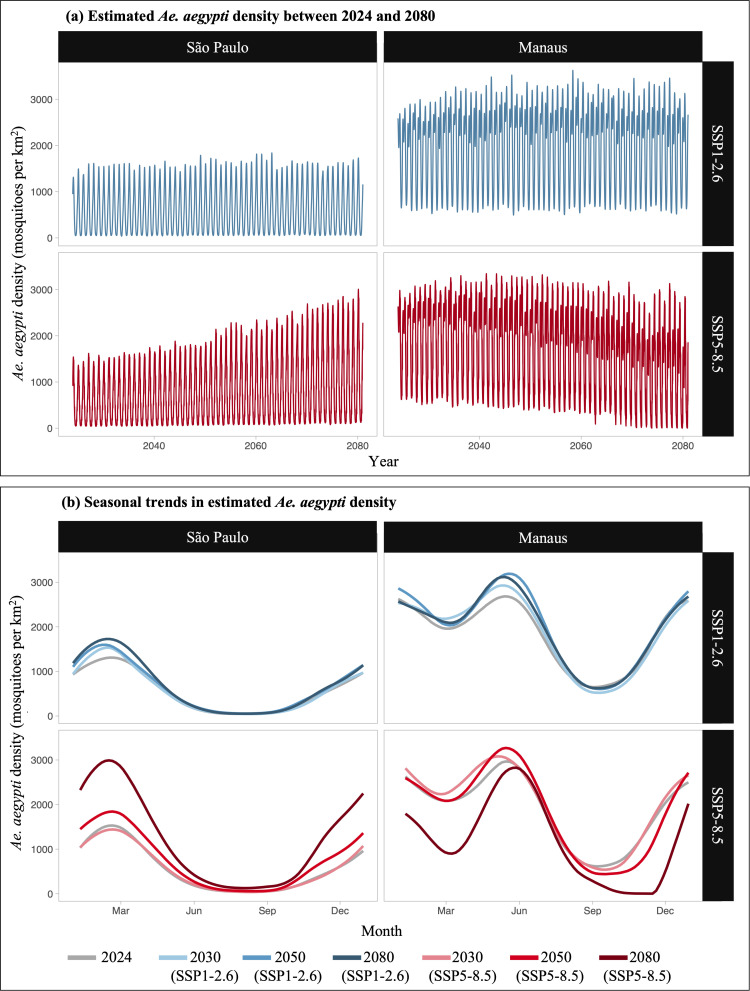
Model-estimated *Ae. aegypti* mosquito density (mosquitoes per km^2^) in São Paulo and Manaus for two future greenhouse gas emission scenarios: SSP1-2.6 (low emissions, strong mitigation) and SSP5-8.5 (high emissions, limited mitigation). Subfigure (a) displays model-estimated density between 2024 and 2080. Subfigure (b) compares the annual trend in density between different years.

Diverse seasonal patterns in *Ae. aegypti* dynamics were projected across Brazil’s climatic regions. Two contrasting examples – the cities of São Paulo and Manaus – are shown in Fig 3. In São Paulo, which has a humid, subtropical climate, *Ae. aegypti* populations were predicted to be highly seasonal, with lower densities during colder, drier months (April to September) and peaks during the warmer, wetter months. Under the low-emission scenario SSP1-2.6, these seasonal trends and peak densities remained consistent. However, under the high-emission scenario SSP5-8.5, peak population sizes steadily increased over time.

In contrast, Manaus, characterised by a humid, tropical monsoon climate, was projected to sustain an endemic *Ae. aegypti* population under the low-emission scenario SSP1-2.6. However, under the high-emission scenario SSP5-8.5, seasonal dynamics were projected to shift, with population crashes occurring between September and December.

### Model predictions validated against existing time-series data

**Fig 4:**
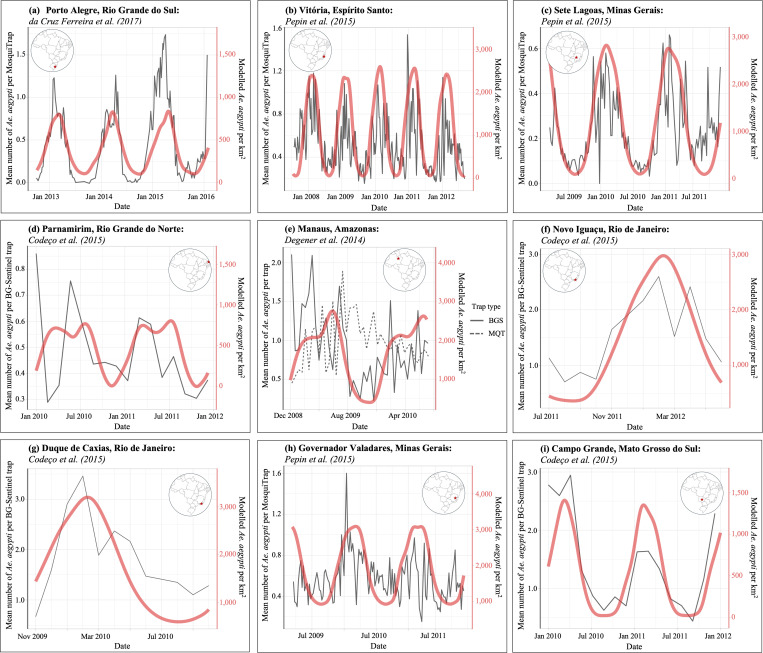
Comparison of published *Ae. aegypti* trap data (grey lines) with model-estimated *Ae. aegypti* density (mosquitoes per km^2^) at nine locations across Brazil. Model estimates and published data were significantly correlated for seven of the nine locations (p < 0.05, modified Chelton method), but not for Parnamirim (subfigure (d); p = 0.32) or Manaus (subfigure (e); p = 0.17 for BG-Sentinal trap data and p = 0.28 for MosquiTrap data). Published data were digitised from Degener et al. (2014), Codeço et al. (2015), da Cruz Ferreira et al. (2017), and Pepin et al. (2015) using WebPlotDigitizer [24–27,39]. All published data were licensed under CC BY 4.0 (https://creativecommons.org/licenses/by/4.0/), permitting reuse of content with appropriate credit and sourcing of original authors.

Time series data at seven of the nine locations were significantly correlated with model predictions (p < 0.05, modified Chelton method). Comparisons of model-predicted *Ae. aegypti* densities and longitudinal field data for all nine locations are presented in Fig4, with Fig 4d and 4e showing non-significant results. Detailed statistical test outcomes are provided in S10 Table.

### Increased vulnerability to mosquito-borne disease outbreaks

**Fig 5:**
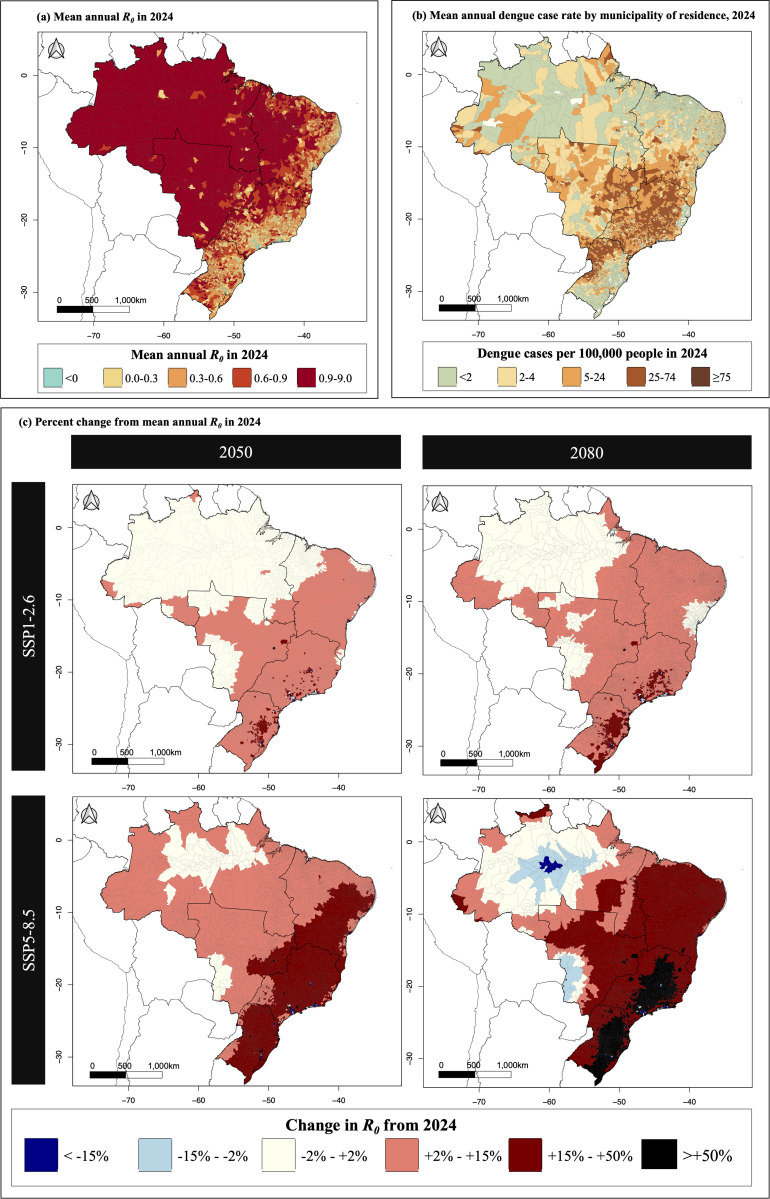
Model-estimated *R*_*0*_ of dengue fever using a mosquito-borne disease transmission model and estimates *Ae. aegypti* mosquito density by municipality in Brazil. Subfigure (a) shows the mean annual model-estimated *R*_*0*_ of dengue fever. Subfigure (b) shows the annual reported dengue fever case rate (cases per 100,000 people) by municipality of residence for 2024. These data were extracted from DATASUS, the health data department of Brazil’s Ministry of Health, via its open data portal (https://datasus.saude.gov.br). These data are publicly available and fall under an open government data policy compatible with the CC BY 4.0 license. Subfigure (c) shows the model-estimated change in mean annual *R*_*0*_ of dengue fever from 2024. Results are presented for, from left to right, years 2050 and 2080, and from top to bottom, two future greenhouse gas emission scenarios: SSP1-2.6 (low emissions, strong mitigation) and SSP5-8.5 (high emissions, limited mitigation). National borders are based on shapefiles from geoBoudaries (https://www.geoboundaries.org/), which are provided under the CC BY 4.0 license [37]. Municipal borders are based on shapefiles from the Instituto Brasileiro de Geografia e Estatística (IBGE), which are available on the Humanitarian Data Exchange (HDX) platform (https://data.humdata.org/dataset/cod-ab-bra) and are provided under the CC BY-IGO license (https://creativecommons.org/licenses/by/3.0/igo/). No changes were made to the IBGE shapefiles.

An epidemiological analysis was undertaken as a conceptual demonstration of the use of our ecological model projections. The mean annual basic reproductive number, *R*_*0*_, for a vector-borne disease such as dengue fever as calculated from model-predicted *Ae. aegypti* abundance is shown in Fig 5a. Higher *R*_*0*_ values indicate greater susceptibility of the population to disease outbreaks. North and Central-west Brazil were predicted to have the highest *R*_*0*_ values.

Predicted future changes in *R*_*0*_ are shown Fig 5c. By 2050, the mean annual *R*_*0*_ was predicted to increase in 99% of municipalities for all SSPs. Under SSP1-2.6, the mean annual *R*_*0*_ increased by between 15% and 50% in 5% of municipalities, whilst under SSP5-8.5, this increase was seen in 62% of municipalities. By 2080, a > 50% increase in *R*_*0*_ was projected in just 1% of municipalities under SSP1-2.6, compared to 28% under SSP5-8.5. The most significant increases in *R*_*0*_ were predicted to occur in Southeast Brazil, a highly populous region.

## Discussion

This study aimed to predict future changes in *Ae. aegypti* mosquito density across Brazil under four climate change scenarios and evaluate the epidemiological implications. Using a biologically structured model sensitive to both climate and anthropogenic factors, we projected spatiotemporal patterns in mosquito density until 2080 under four SSPs. While previous studies have shown that climate change is likely to increase temperature suitability for mosquitoes, our study adds three key findings: (i) mosquito density is projected to rise nationally in Brazil but not uniformly, with regional variation in magnitude and timing, and some areas exceeding thermal limits; (ii) Southeast and South Brazil could experience the most substantial relative increases; and (iii) these shifts are likely to expand disease transmission potential, even in areas currently considered lower risk [1,30].

A major contribution of this work is its biologically explicit modelling approach, which incorporates non-linear climate effects on mosquito development, urbanisation patterns, and human population growth. This approach goes beyond occurrence probability models to generate density estimates that can be directly linked to epidemiological risk via *R*_*0*_, offering an actionable basis for public health planning. The model is applied at high spatial and temporal resolution using Brazil-specific entomological, demographic, and climate data, producing projections aligned with subnational administrative boundaries. This granularity offers a valuable tool for informing both national policies and local public health planning.

Our results demonstrate that the future burden of mosquito-borne diseases in Brazil is strongly shaped by climate policy trajectories. Under the highest-emission, unmitigated warming scenario (SSP5-8.5), mosquito densities were projected to nearly double by 2080 in southern states. In contrast, under SSP1-2.6, which reflects a scenario where climate policies successfully limit global warming to below 2°C, national mosquito density was projected to rise by 11%, and the greatest regional density was 21% in South Brazil. These stark differences underscore the scale of the potential public health threat and the extent to which it hinges on climate action, particularly in countries like Brazil, where climate and urbanisation already create high baseline risks for arboviral diseases and have already contributed to expansion of dengue transmission [6].

Importantly, our analysis shows that intermediate scenarios – SSP2-4.5 (stabilisation with moderate emissions reductions) and SSP3-7.0 (fragmentation, limited international cooperation and low climate mitigation) – also yield meaningful increases in both mosquito abundance and disease risk. For instance, national mosquito density was projected to increase by 21% and 32%, respectively, under SSP2-4.5 and SSP3-7.0 by 2080. These trajectories suggest that even moderate climate inaction could leave vast regions of Brazil vulnerable to intensified arboviral transmission, and thus deserve focussed attention in adaptation planning.

Our findings complement existing studies by similarly showing increased thermal suitability for *Ae. aegypti*, expanded seasonal windows and increased human population exposure, particularly under higher-emissions scenarios [1,30]. Further, our findings reinforce the nuance that, while overall risk grows, some regions may face diminishing thermal suitability for *Ae. aegypti* due to excessive heat, as demonstrated by the changing seasonal trends in Manaus (Fig 3).

Populous Southeast Brazil was project to see the largest increases in *R*_*0*_ for dengue fever, driven by mosquito populations growing faster than human populations. In contrast, rural and disadvantaged regions in the North and Central areas, though showing smaller *R₀* increases, remain highly vulnerable due to high vector-to-host ratios, which are compounded with limited public health infrastructure and existing social disparities [31]. These compounded risks highlight the need for targeted, region-specific interventions. Higher *R*_*0*_ in rural areas aligns with findings from Vietnam and Malaysia, where similar dynamics drive dengue risk [32,33]. However, mismatches between *R*_*0*_ and reported dengue cases (Fig 5) likely reflect gaps in surveillance; studies have estimating up to 17 unreported cases for every reported case in Brazil [34,35]. Addressing these intersecting challenges will require tailored strategies that combine climate mitigation, urban planning, and strengthened public health systems.

Our study offers three key methodological advances. First, a biologically structured model accounted for non-linear climate effects on *Ae. aegypti*, including the detrimental impacts of extreme temperatures on mosquito life history traits [4,5,13]. Second, incorporation of anthropogenic factors like urbanisation and population growth enabled a spatiotemporal analysis of mosquito density that captures human-mosquito interactions. Third, by estimating mosquito density rather than occurrence probability, we link ecological changes to epidemiological outcomes (e.g., *R*_*0*_ of dengue fever). This approach directly connects emissions reductions to decreases in mosquito density, underscoring the public health relevance of climate action.

Validation against historical data showed strong model accuracy in seven of nine tested locations (Fig 4). The two sites without significant correlations had much sparser trapping (24 traps vs. up to 1,392, see S10 Table), likely increasing data variability and reducing alignment. Despite this, visual comparisons (e.g., Parnamirim, Fig 4d) reveal notable similarities, suggesting that data limitations rather than model shortcomings may go some way to explain discrepancies.

While our study offers valuable insights into climate impacts on *Ae. aegypti* populations and disease risk, a critical caveat is that our model does not include future advances in public health infrastructure, vector control, or biomedical interventions. This may overestimate future risk, as emerging tools like *Wolbachia*-infected mosquitoes, gene drives, next-generation vaccines, and improvements in housing and sanitation could significantly reduce transmission. Future models should incorporate these intervention scenarios to better capture the dynamic interactions between ecology, health systems, and climate adaptation.

Further, our epidemiological model does not include factors such as human age structure, mosquito dispersal, immunity, or viral genetics, which can affect transmission. A fully comprehensive model is beyond the scope of this paper, however, using a simplified approach, our results have demonstrated the epidemiological application our ecological model. We focussed solely on *Ae. aegypti*, potentially overlooking other vectors like *Ae. albopictus*. Additionally, our method uses infestation indices as proxies for *Ae. aegypti* occurrence; while effective for indicating mosquito presence, these cross-sectional indices do not directly measure arboviral disease risk [36]. Finally, reliance on climate and populations projections, which have their own methodological limitations, may introduce further uncertainty.

This study illustrates the dual threat posed by climate change: the exacerbation of existing public health challenges and the creation of new vulnerabilities in Brazil’s mosquito-borne disease landscape. By linking mosquito abundance and disease risk to specific emission pathways, we show that climate action can substantially reduce future disease burden. For example, shifting from SSP5–8.5 to SSP1–2.6 could cut projected mosquito density increases from 31% to 11% nationally, and from 92% to 17% in Southeast Brazil by 2080, providing a tangible target for climate and health policy. As climate change accelerates, effective control of mosquito-borne diseases will hinge on integrated approaches that couple emissions reduction with proactive public health strategies. Strengthening surveillance and forecasting systems, alongside closing infrastructure and healthcare gaps in underserved areas, will be vital to safeguarding vulnerable populations and enhancing future resilience.

## Supporting information

S1 Text Derivation of survival equations. (PDF)

S2 Text Statistical software and packages employed. (PDF)

S3 Text Temperature variation. (PDF)

S4 Text Juvenile density dependence. (PDF)

S5 Text LIRAa sampling protocol. (PDF)

S6 Text Boosted Regression Trees (BRTs). (PDF)

S7 Text Modified Chelton method for comparison of time series. (PDF)

S8 Text Calculation of *R*_*0.*_ (PDF)

S1 Table Parameter values and 95% confidence intervals for functions describing the relationships between temperature and *Ae. aegypti* life history traits. (PDF)

S2 Table Details of datasets used throughout the study and their sources. (PDF)

S3 Table Parameter values and their source for calculation of *R*_*0*_ for dengue fever. (PDF)

S4 Table Model-estimated mean annual Ae. aegypti density (mosquitoes per km²) in Brazil’s ten largest cities. (PDF)

S5 Table Model-estimated mean annual Ae. aegypti density (mosquitoes per km²) in Brazil’s five geographical regions. (PDF)

S6 Table Model-estimated mean annual Ae. aegypti density (mosquitoes per km²) in Brazil’s 27 states. (PDF)

S7 Table Model-estimated fold-change in mean annual Ae. aegypti density (mosquitoes per km²) from 2024 in Brazil’s ten largest cities. (PDF)

S8 Table Model-estimated fold-change in mean annual Ae. aegypti density (mosquitoes per km²) from 2024 in Brazil’s five geographical regions. (PDF)

S9 Table Model-estimated fold-change in mean annual Ae. aegypti density (mosquitoes per km²) from 2024 in Brazil’s 27 states. (PDF)

S10 Table Details of temporal data of *Ae. aegypti* used to validate model predictions. (PDF)
