# Supplementary material for: Climate change, urbanisation and transmission potential: Aedes aegypti mosquito projections forecast future arboviral disease hotspots in Brazil
Source: PLoS Negl Trop Dis. 2025 Sep 18;19(9):e0013415. doi: 10.1371/journal.pntd.0013415 (PMC12445552; doi:10.1371/journal.pntd.0013415)
Supplement: S1 Text — (PDF) [file pntd.0013415.s001.pdf]

S1 Text: Derivation of survival equations

The instantaneous, stage specific survival can be written as:

$$S_i(t) = e^{-\int_{t-\tau_i(t)}^{t} \mu_i(t)dt} \qquad \text{(Equation A)}$$

where $i = \{J, A\}$, J and A denote juvenile and adult mosquito populations, respectively, $\mu_i(t)$ is the stage-specific mortality and τ_i is the stage-specific development time. First, the derivative of Equation A is taken to give:

$$\frac{dS_i(t)}{dt} = S_i(t)\left[\frac{d}{dt}\left(-\int_{t-\tau_i(t)}^{t} \mu_i(t)\right)\right] \qquad \text{(Equation B)}$$

which is further evaluated as:

$$\frac{dS_i(t)}{dt} = S_i(t)\left[\frac{d}{dt}\big(t-\tau_i(t)\big)\mu_i\big(t-\tau_i(t)\big) - \frac{dt}{dt}\mu_i(t)\right] \qquad \text{(Equation C)}$$

and subsequently as:

$$\frac{dS_i(t)}{dt} = S_i(t)\left[\left(1-\frac{d\tau_i(t)}{dt}\right)\mu_i\big(t-\tau_i(t)\big) - \mu_i(t)\right] \qquad \text{(Equation D)}$$

Since ${d\tau_i(t)}/{dt} = 1 - \left({g_i(t)}/{g_i(t-\tau_i(t))}\right)$, then this can be substituted into Equation D to give:

$$\frac{dS_i(t)}{dt} = S_i(t)\left[\left(1-1-\frac{g_i(t)}{g_i\big(t-\tau_i(t)\big)}\right)\mu_i\big(t-\tau_i(t)\big) - \mu_i(t)\right] \qquad \text{(Equation E)}$$

which simplifies to

$$\frac{dS_i(t)}{dt} = S_i(t)\left[\frac{g_i(t)\mu_i(t-\tau_i(t))}{g_i(t-\tau_i(t))} - \mu_i(t)\right]. \qquad \text{(Equation F)}$$
