# Supplementary material for: Climate change, urbanisation and transmission potential: Aedes aegypti mosquito projections forecast future arboviral disease hotspots in Brazil
Source: PLoS Negl Trop Dis. 2025 Sep 18;19(9):e0013415. doi: 10.1371/journal.pntd.0013415 (PMC12445552; doi:10.1371/journal.pntd.0013415)
Supplement: S2 Text — (PDF) [file pntd.0013415.s002.pdf]

S2 Text: Statistical software and packages employed

All analyses were conducted using R version 4.0 [1]. Delay-differential equations were solved using the dede() function in the deSolve package [1,2]. Daily temperature and precipitation estimates were derived from each monthly data point for the Tier 1 scenarios SSP1-2.6, SSP2-4.5, SSP3-7.0 and SSP5-8.5 using cubic spline interpolation with the splint() function from the fields package [3]. *Aedes aegypti* population density estimates were spatially smoothed using fixed rank kriging in the FRK package for R version 4.0 [4]. Boosted Regression Trees (BRTs) were implemented using the gbm.step() from the function from the dismo package [5].

References

1. R Core Team. R: A Language and Environment for Statistical Computing. Vienna, Austria; 2018.
2. Soetaert K, Petzoldt T, Setzer RW. Solving Differential Equations in *R* : Package deSolve. J Stat Softw. 2010;33. doi:10.18637/jss.v033.i09
3. Nychka D, Furrer R, Paige J, Sain S. fields: Tools for spatial data. Boulder, CO, USA: University Corporation for Atmospheric Research; 2021. Available: https://github.com/dnychka/fieldsRPackage
4. Zammit-Mangion A, Cressie N. FRK : An *R* Package for Spatial and Spatio-Temporal Prediction with Large Datasets. J Stat Softw. 2021;98. doi:10.18637/jss.v098.i04
5. Hijmans RJ, Phillips S, Leathwick J. dismo: Species Distribution Modeling. R package version 1.3-15. Available: https://github.com/rspatial/dismo
