# Supplementary material for: Climate change, urbanisation and transmission potential: Aedes aegypti mosquito projections forecast future arboviral disease hotspots in Brazil
Source: PLoS Negl Trop Dis. 2025 Sep 18;19(9):e0013415. doi: 10.1371/journal.pntd.0013415 (PMC12445552; doi:10.1371/journal.pntd.0013415)
Supplement: S3 Text — (PDF) [file pntd.0013415.s003.pdf]

**S3 Text: Temperature variation**

As noted in the main text, Mordecai et al. (2017) fitted thermal response models to empirical data for *Ae. aegypti* and *Aedes albopictus*, defining adult fecundity, $b(t)$, using a Brière function (see Equation 8 in main text) [1]. Mordecai et al. (2017) also provided fitted functions for adult longevity and juvenile development rate, $g_J(t)$, but Brière and quadratic models would produce negative values at certain temperatures within our study range. Therefore, we fitted alternative functions for $\mu_A(t)$ and $g_J(t)$ to empirical data extracted from the literature across a spectrum of temperatures. References for studies from which data were extracted are given in S1 Table.

A Gaussian function was fitted to adult longevity data using non-linear least squares regression (see Equation 9 in main text). The p-value was <0.001 for all parameters. Following application of Equation 9, if $b(t) < 0$ or $b(t) \rightarrow \infty$, then $b(t)$ was truncated at zero.

A 2$^{nd}$-degree polynomial was fitted to juvenile development time (see Equation 10 in main text), which was a significantly better fit than a linear function ($p$<0.001, likelihood ratio test (LRT)).

To derive a temperature-dependent function for juvenile mortality, $\mu_J(t)$, it was first defined by rearranging Equation 6 (see main text), which describes juvenile survival:

$$\mu_J(t) = \frac{ln\left(S_J(t)\right)}{\tau_J(t)} \qquad \text{( Equation A )}$$

For the purposes of parameter estimation, it was assumed that $\tau_J(t) = \tau_J(t-1)$ and $\delta_J(t)J(t) \approx 0$. To satisfy Equation A, $\tau_J(t)$ was defined according to Equation 10 (see main text), and based on Mordecai et al. (2017), juvenile survival was estimated using the following quadratic function:

$$S_J(t) = -c_S(T(t) - T_{0S})(T(t) - T_{MS}) \qquad \text{( Equation B )}$$

$T_{0S}$ and $T_{MS}$ are the thermal minimum and maximum respectively and $c_S$ is a numeric constant.

Using Equations 10 and B, $S_J(t)$ and $\tau_J(t)$ were estimated for $T = 1,2, \ldots ,50$. Values of $S_J(t)$ from Equation B were truncated at zero. Juvenile mortality, $\mu_J(t)$, was subsequently calculated for $T = 1,2, \ldots ,50$ using Equation A. When $S_J(t) = 0$, then $\mu_J(t) \rightarrow \infty$. A 4$^{th}$-degree polynomial was fitted to the resulting estimates of $\mu_J(t)$ using non-linear least-squares regression. This 4$^{th}$-degree polynomial was a significantly better fit than 2$^{nd}$- and 3$^{rd}$-degree polynomials ($p$<0.001 for both, LRT), and is defined in Equation 11 in the main text.

**References**


1. Mordecai EA, Cohen JM, Evans M V, Gudapati P, Johnson LR, Lippi CA, et al. Detecting the impact of temperature on transmission of Zika, dengue, and chikungunya using mechanistic models. PLoS Negl Trop Dis. 2017;11: e0005568. doi:10.1371/journal.pntd.0005568
