# Supplementary material for: Climate change, urbanisation and transmission potential: Aedes aegypti mosquito projections forecast future arboviral disease hotspots in Brazil
Source: PLoS Negl Trop Dis. 2025 Sep 18;19(9):e0013415. doi: 10.1371/journal.pntd.0013415 (PMC12445552; doi:10.1371/journal.pntd.0013415)
Supplement: S4 Text — (PDF) [file pntd.0013415.s004.pdf]

S4 Text: Juvenile density dependence

We defined density-dependent juvenile mortality as a function of existing juvenile density and precipitation, with mortality increasing under conditions of high density and low precipitation. This approach aligns with established methodologies in mosquito life-cycle modelling, such as Ewing et al. (2016), who employed a predator-prey relationship to model elevated larval mortality at high larval densities [1]. By incorporating precipitation as a modulator, we provide a biologically realistic representation of environmental constraints on juvenile survival.

Rainfall was assumed to enhance the environment's carrying capacity by creating and maintaining oviposition sites. Exponential weighting was chosen because it has been shown to better fit entomological field data than linear weighting [2]. White et al. (2011) applied this model to *Anopheles gambiae* and found an optimal ω of 4 days (95% CI: 2.5–7). However, we acknowledge that extremely heavy rainfall may saturate or wash out breeding sites, a non-linear effect documented in field studies that is not explicitly captured in our current model but may warrant future investigation to improve ecological realism [3].

Our initial modelling indicated that temperature alone was insufficient to prevent unchecked mosquito population growth, highlighting the necessity of incorporating additional ecological mechanisms to accurately capture population dynamics. Various mechanisms of density dependence in mosquito larvae have been proposed, including resource limitation, chemical interference, and cannibalism [4–6]. While a detailed exploration of these mechanisms lies beyond the scope of this study, we employed the parameter λ to regulate carrying capacity as influenced by precipitation, effectively capturing the impact of density-dependent mortality within a realistic ecological framework.

Sensitivity analysis on parameter λ revealed that variations in its value affected the absolute upper limit of predicted *Ae. aegypti* density but had minimal impact on temporal and spatial trends or the relative ratios of density between time points and locations. Importantly, this robustness ensures that our core results, particularly the projected percentage increases in *Ae. aegypti* density, remain valid across a reasonable range of λ values. These findings underscore the reliability of our model outputs for identifying trends and relative changes in mosquito density, regardless of minor uncertainties in parameter specification.

References

1. Ewing DA, Cobbold CA, Purse B V, Nunn MA, White SM. Modeling the effects of temperature on the seasonal population dynamics of temperate mosquitoes. J Theor Biol. 2016;400: 65–79.

2. White MT, Griffin JT, Churcher TS, Ferguson NM, Basáñez M-G, Ghani AC. Modelling the impact of vector control interventions on Anopheles gambiae population dynamics. Parasit Vectors. 2011;4: 153. doi:10.1186/1756-3305-4-153

3. Koenraadt CJM, Harrington LC. Flushing effect of rain on container-inhabiting mosquitoes Aedes aegypti and Culex pipiens (Diptera: Culicidae). J Med Entomol. 2008;45: 28–35. doi:10.1603/0022-2585(2008)45[28:feoroc]2.0.co;2

4. Porretta D, Mastrantonio V, Crasta G, Bellina R, Comandatore F, Rossi P, et al. Intra-instar larval cannibalism in *Anopheles gambiae* (s.s.) and *Anopheles stephensi* (Diptera: Culicidae). Parasit Vectors. 2016;9.

5. Dye C. Competition amongst larval *Aedes aegypti*: the role of interference. Ecol Entomol. 9: 355–357.

6. Couret J, Dotson E, Benedict MQ. Temperature, larval diet, and density effects on development rate and survival of *Aedes aegypti* (Diptera: Culicidae). PLoS One. 2014;9: e87468.
