# Supplementary material for: Climate change, urbanisation and transmission potential: Aedes aegypti mosquito projections forecast future arboviral disease hotspots in Brazil
Source: PLoS Negl Trop Dis. 2025 Sep 18;19(9):e0013415. doi: 10.1371/journal.pntd.0013415 (PMC12445552; doi:10.1371/journal.pntd.0013415)
Supplement: S5 Text — (PDF) [file pntd.0013415.s005.pdf]

S5 Text. LIRAa sampling protocol

Ae. aegypti occurrence data used in this study come from comprehensive entomological surveillance conducted by the Brazilian Ministry for Health. The sampling methodology followed the LIRAa protocol which is described by the Brazilian Ministry of Health [1–3]. LIRAa is used in all municipalities regardless of size. The sampling methodology for each municipality is determined according to the municipality population density and the number of buildings. Urban areas within municipalities were divided into strata with comparable socioeconomic characteristics to achieve homogeneity in each stratum. Strata have between 8,100-12,000 premises each depending on the municipality population density.

Samples were collected using a cluster sampling approach with blocks as a primary unit of sampling and buildings as a secondary unit. One can imagine LIRAa as establishing a spatial mesh with a block to be inspected at each intersection. Within the sampling block, buildings are inspected. Each block falls within a stratum. An independent sample is drawn from within each block, with the sample size being determined by the stratum size. In this way, LIRAa controls well for sampling effort.

The LIRAa protocol generates various indices describing levels of mosquito infestation [1–3]. We used the building index – the proportion of buildings infested – as a response variable to model *Ae. aegypti* occurrence probability due to spatial heterogeneity in anthropogenic variables.

References

1. Secretaria de Vigilância Sanitária em Saúde. Diagnóstico rápido nos municípios para vigilância entomológica do *Aedes aegypti* no Brasil - LIRAa. Metodologia para avaliação dos índices de Breteau e predial. Brasília; 2005.

2. Coelho GE. Challenges in the control of *Aedes aegypti*. Rev Inst Med Trop Sao Paulo. 2012;54: 13–14.

3. Coelho RG, Lourenço-de-Oliveira R, Braga IA. Updating the geographical distribution and frequency of *Aedes albopictus* in Brazil with remarks regarding its range in the Americas. Mem Inst Oswaldo Cruz. 2014;109: 787–796.
