# Supplementary material for: Climate change, urbanisation and transmission potential: Aedes aegypti mosquito projections forecast future arboviral disease hotspots in Brazil
Source: PLoS Negl Trop Dis. 2025 Sep 18;19(9):e0013415. doi: 10.1371/journal.pntd.0013415 (PMC12445552; doi:10.1371/journal.pntd.0013415)
Supplement: S6 Text — (PDF) [file pntd.0013415.s006.pdf]

S6 Text. Boosted Regression Trees (BRTs)

Boosted Regression Trees (BRTs) are particularly effective for mapping mosquito distribution and mosquito-borne diseases due to their ability to model complex, non-linear relationships between predictors [1–3]. These models iteratively refine regression trees using gradient boosting, minimizing the unexplained variation in the response variable with each new tree.

BRTs were fitted using the gbm.step() function from the dismo package in R version 4.0, with default hyperparameters (tree complexity = 4, learning rate = 0.005, bag fraction = 0.75, step size = 10, and cross-validation folds = 10).[4,5] Each model underwent ten-fold cross-validation, where the dataset was divided into ten subsets. Nine subsets were used for training, and predictive accuracy was assessed on the remaining data based on predictive deviance. A total of 100 cross-validated models were fitted, and the mean of these models was calculated to account for variability.

The trained BRT model was then used to project *Ae. aegypti* occurrence probability at a 5 km^2 resolution. Population projections and urban accessibility data were adjusted to estimate occurrence probabilities across different time periods and SSPs, with all covariates extracted at a 5 km^2 resolution. For future projections, climatic variables were not used to estimate *Ae. aegypti* occurrence probability; instead, temperature was fixed at 26°C to isolate the effects of anthropogenic variables. This allowed the BRT model to assess the impact of human-driven factors on mosquito distribution, while temperature-induced changes in *Ae. aegypti* density were already accounted for in the life-history model.

References

1. Bhatt S, Gething PW, Brady OJ, Messina JP, Farlow AW, Moyes CL, et al. The global distribution and burden of dengue. Nature. 2013;496: 504–7. doi:10.1038/nature12060

2. Kraemer MUG, Reiner RC, Brady OJ, Messina JP, Gilbert M, Pigott DM, et al. Past and future spread of the arbovirus vectors Aedes aegypti and Aedes albopictus. Nat Microbiol. 2019;4: 854–863. doi:10.1038/s41564-019-0376-y

3. Messina JP, Brady OJ, Golding N, Kraemer MUG, Wint GRW, Ray SE, et al. The current and future global distribution and population at risk of dengue. Nat Microbiol. 2019;4: 1508–1515. doi:10.1038/s41564-019-0476-8

4. Hijmans RJ, Phillips S, Leathwick J. dismo: Species Distribution Modeling. R package version 1.3-15. Available: https://github.com/rspatial/dismo

5. R Core Team. R: A Language and Environment for Statistical Computing. Vienna, Austria; 2018.
