# Supplementary material for: Climate change, urbanisation and transmission potential: Aedes aegypti mosquito projections forecast future arboviral disease hotspots in Brazil
Source: PLoS Negl Trop Dis. 2025 Sep 18;19(9):e0013415. doi: 10.1371/journal.pntd.0013415 (PMC12445552; doi:10.1371/journal.pntd.0013415)
Supplement: S7 Text — (PDF) [file pntd.0013415.s007.pdf]

S7 Text. Modified Chelton method for comparison of time series

A literature search identified entomological time series data from locations in Brazil to which model estimates could be compared. Data from supplementary materials or appendices were used when available; otherwise, plotted data were digitized with WebPlotDigitizer [1]. Studies on vector control or those using only ovitraps were excluded. For datasets with multiple studies or traps, the one with the longest time span was used, and data from multiple traps were averaged.

The modified Chelton method is outlined by Pyper and Peterman (1998) [2]. In brief, Pyper and Peterman adjusted Chelton's 1984 method by specifying $N^* - 2$ rather than N^* degrees of freedom [3]. Pyper and Peterman (1998) observed that this approach offered improved balance between N^* and Type I error rates [2]. The degrees of freedom, N^*, was calculated by rearrangement of Equation (1) in Pyper and Peterman (1998) to give

$$N^* = \frac{N}{1+2\sum_{j=1}^{\infty}\left(\frac{N-j}{N}p_{xx}(j)p_{yy}(j)\right)} - 2 \qquad \text{(Equation A)}$$

where N is the sample size and $p_{xx}(j)$ and $p_{yy}(j)$ are the autocorrelations of two time series, x and y, at a lag, j. T-statistics were calculated by

$$t = r_{xy}\sqrt{\frac{N^*}{1-r_{xy}{}^2}} \qquad \text{(Equation B)}$$

where r_{xy} is the correlation between two time series, x and y. P-values were extracted from the t-statistics.

References

1. Rohatgi A. WebPlotDigitizer. [cited 6 Feb 2025]. Available: https://automeris.io/
2. Pyper BJ, Peterman RM. Comparison of methods to account for autocorrelation in correlation analyses of fish data. Canadian Journal of Fisheries and Aquatic Science. 1998;55.
3. Chelton DB. Commentary: short-term climatic variability in the Northeast Pacific Ocean. In: Pearcy W, editor. The influence of ocean conditions on the production of salmonids in the North Pacific. Corvallis, Oregon: Oregon State University Press; 1984. pp. 87–99.
