# Supplementary material for: Climate change, urbanisation and transmission potential: Aedes aegypti mosquito projections forecast future arboviral disease hotspots in Brazil
Source: PLoS Negl Trop Dis. 2025 Sep 18;19(9):e0013415. doi: 10.1371/journal.pntd.0013415 (PMC12445552; doi:10.1371/journal.pntd.0013415)
Supplement: S8 Text — (PDF) [file pntd.0013415.s008.pdf]

S8 Text. Calculation of R_0

The R_0 of the two-compartment Ross-Macdonald model for dengue fever presented in Equations 13-14 in the main text can be determined as the determinant of the Jacobian matrix of the linearised system. From Equations 13-14, we can write the Jacobian as

$$R_0 = Det\begin{bmatrix} \frac{\partial H}{dH} & \frac{\partial H}{dV} \\ \frac{\partial V}{dH} & \frac{\partial V}{dV} \end{bmatrix} \qquad \text{(Equation A)}$$

Then, calculating the partial derivatives:

$$R_0 = Det\begin{bmatrix} aBV(t)\frac{A^*}{2N^*} - r & aB\frac{A^*}{2N^*}\big(1 - H(t)\big) \\ aC\big(1 - V(t)\big) & aCH(t) - \mu_A{}^* \end{bmatrix} \qquad \text{(Equation B)}$$

Assuming that dengue invades an entirely healthy population, $H(t) \rightarrow 0$ and $V(t) \rightarrow 0$. The adult *Ae. aegypti* population, $A(t)$, human population, $N(t)$, and adult *Ae. aegypti* mortality, $\mu_A(t)$, are assumed to be at equilibrium ($A(t) = A^*$, $N(t) = N^*$ and $\mu_A(t) = \mu_A{}^*$). Therefore R_0 is described by

$$R_0 = Det\begin{bmatrix} -r & \frac{aBA^*}{2N^*} \\ aC & -\mu_A{}^* \end{bmatrix} \qquad \text{(Equation C)}$$
