# Supplementary material for: Climate change, urbanisation and transmission potential: Aedes aegypti mosquito projections forecast future arboviral disease hotspots in Brazil
Source: PLoS Negl Trop Dis. 2025 Sep 18;19(9):e0013415. doi: 10.1371/journal.pntd.0013415 (PMC12445552; doi:10.1371/journal.pntd.0013415)
Supplement: S1 Table — (PDF) [file pntd.0013415.s009.pdf]

S1 Table. Parameter values and 95% confidence intervals for functions describing the relationships between temperature and *Ae. aegypti* life history traits. Functional forms are shown in Equations 8-11 in the main text. It should be noted that for parameters taken for Mordecai et al. (2017) (adult fecundity and juvenile survival), 95% credible intervals are given due to the Bayesian framework used to derive parameters. 95% confidence intervals are not given for juvenile mortality because juvenile mortality was calculated using Equation A in S3 Text before fitting a polynomial function. The variability in $\mu_J(t)$ is therefore captured by the 95% confidence intervals on parameters describing $S_J(t)$ and $\tau_J(t)$. Where parameters are determined by equations in the main text, this is denoted by "Eq." followed by the equation number as used in the main text.

Trait	Parameter	Value	95% CI		Source
			2.5%	*97.5%*	
Adult fecundity, b	c_b	8.56×10^{-3}	3.78×10^{-3}	1.41×10^{-2}	Reference [1] & Eq. 8
	T_{0b}	14.58	8.08	20.60	
	T_{Mb}	34.61	34.00	35.77	
Adult mortality, μ_A	k	36.55	32.27	23.85	References [2–10] & Eq. 9
	γ	25.17	23.85	26.50	
	σ	7.31	5.90	8.71	
Juvenile development time, $\tau_J(t)$	β_{0g}	133.53	113.45	153.61	References [2,7,9–18] & Eq. 10
	β_{1g}	-8.29	-9.90	-6.69	
	β_{2g}	0.14	0.10	0.17	
Juvenile survival, S_J	c_S	5.99×10^{-3}	6.82×10^{-3}	5.13×10^{-3}	Reference [1] & Eq. B in S3 Text
	T_{0S}	13.56	12.56	14.51	
	T_{MS}	38.29	37.54	39.02	
Juvenile mortality, μ_J	$\beta_{0\mu}$	3.37×10^{-3}	-	-	Eq.11
	$\beta_{1\mu}$	-5.88×10^{-2}	-	-	
	$\beta_{2\mu}$	4.05×10^{-3}	-	-	
	$\beta_{3\mu}$	-1.26×10^{-4}	-	-	
	$\beta_{4\mu}$	1.47×10^{-6}	-	-	
Density-dependent Juvenile mortality, $\delta(t)$	K	Eq. 12			Reference [1] & Eq. 12
	λ	7.5×10^{8}	2.5	7	
	ω	4	-	-	

References

1. Mordecai EA, Cohen JM, Evans M V, Gudapati P, Johnson LR, Lippi CA, et al. Detecting the impact of temperature on transmission of Zika, dengue, and chikungunya using mechanistic models. PLoS Negl Trop Dis. 2017;11: e0005568. doi:10.1371/journal.pntd.0005568

2. Marinho RA, Beserra EB, Bezerra-Gusmão MA, Porto Vde S, Olinda RA, Dos Santos CA. Effects of temperature on the life cycle, expansion, and dispersion of *Aedes aegypti* (Diptera: Culicidae) in three cities in Paraiba, Brazil. Journal of Vector Ecology. 2016;41: 1–10.

3. Muttis E, Balsalobre A, Chuchuy A, Mangudo C, Ciota AT, Kramer LD, et al. Factors related to *Aedes aegypti* (Diptera: Culicidae) populations and temperature determine differences on life-history traits with regional implications in disease transmission. J Med Entomol. 2018;55: 1105–1112.

4. Chadee DD, Martinez R, Sutherland JM. *Aedes aegypti* (L.) mosquitoes in Trinidad, West Indies: longevity case studies. Journal of Vector Ecology. 2017;42: 130–135.

5. Goindin D, Delannay C, Ramdini C, Gustave J, Fouque F. Parity and longevity of *Aedes aegypti* according to temperatures in controlled conditions and consequences on dengue transmission risks. PLoS One. 2015;10.

6. Beeuwkes H, Kerr JA, Weathersbee AA, Taylor AW. Observations on the bionomics and comparative prevalence of the vectors of yellow fever and other domestic mosquitoes of West Africa, and the

epidemiological significance of seasonal variations. Transactions of the Royal Society of Tropical Medicine and Hygeine. 1933;26: 425–447.

7. Yang HM, Macoris MLG, Galvani KC, Andrighetti MT, Wanderley SM. Assessing the effects of temperature on the population of *Aedes aegypti*, the vector of dengue. Epidemiol Infect. 2009;137: 1188–1211.

8. McMeniman CJ, Lane R V, Cass BN, Fong AW, Sidhu M, Yang YF, et al. Stable introduction of a life-shortening *Wolbachia* infection into the mosquito *Aedes aegypti*. Science (1979). 2009;323: 141–144.

9. Beilhe LB, Delatte H, Juliano SA, Fontenille D, Quilici S. Ecological interactions in *Aedes* species on Reunion Island. Med Vet Entomol. 2012;27: 387–397.

10. Beserra EB. Efeitos da temperatura no ciclo de vida, exigências térmicas e estimativas do número de gerações anuais de *Aedes aegypti* (Diptera, Culicidae). Iheringia Ser Zool. 2009;99: 142–148.

11. Bar-Zeev M. The effect of temperature on the growth rate and survival of the immature stages of *Aedes aegypti* (L.). Bull Entomol Res. 1958;49: 157–163.

12. Couret J, Dotson E, Benedict MQ. Temperature, larval diet, and density effects on development rate and survival of *Aedes aegypti* (Diptera: Culicidae). PLoS One. 2014;9: e87468.

13. Reuda LM, Patel KJ, Axtell RC, Stinner RE. Temperature-dependent development and survival rates of *Culex quinquefasciatus* and *Aedes aegypti* (Diptera: Culicidae). J Med Entomol. 1990;27: 892–898.

14. Tun-Lin W, Burkot TR, Kay BH. Effects of temperature and larval diet on development rates and survival of the dengue vector *Aedes aegypti* in north Queensland, Australia. Med Vet Entomol. 2000;14: 31–37.

15. Kaimura K, Matsuse IT, Takahashi H, Komukai J, Fukuda T, Suzuki K, et al. Effect of temperature on the development of *Aedes aegypti* and *Aedes albopictus*. Medical Entomology and Zoology. 2002;53: 55–58.

16. Shannon RC, Putnam P. The biology of Stegomyia under laboratory conditions. I. The analysis of factors which influence larval development. Proc Entomol Soc Wash. 1934;36: 185–242.

17. Richardson KAA, Hoggmann A, Johnson P, Ritchie S, Kearney MR. Thermal sensitivity of *Aedes aegypti* from Australia: empirical data and prediction of effects on distribution. J Med Entomol. 2011;48: 914–923.

18. Westbrook CJ. Larval ecology and adult vector competence of invasive mosquitoes *Aedes albopictus* and *Aedes aegypti* for chikungunya virus. University of Florida. 2010.
