# Supplementary material for: Climate change, urbanisation and transmission potential: Aedes aegypti mosquito projections forecast future arboviral disease hotspots in Brazil
Source: PLoS Negl Trop Dis. 2025 Sep 18;19(9):e0013415. doi: 10.1371/journal.pntd.0013415 (PMC12445552; doi:10.1371/journal.pntd.0013415)
Supplement: S2 Table — (PDF) [file pntd.0013415.s010.pdf]

S2 Table. Details of datasets used throughout the study and their sources. Data used for validation of model results are not described in this table because they are described in S10 Table.

Dataset	Description	Use	Source
Temperature (°C)	Projected temperature under different future greenhouse gas emission scenarios.	Used as an input for modelling mosquito populations.	Coupled Model Intercomparison Project Phase 6 (CMIP6) [1]
Precipitation (mm day^{-1})	Projected precipitation under different future greenhouse gas emission scenarios.	Used as an input for modelling mosquito populations.	Coupled Model Intercomparison Project Phase 6 (CMIP6) [1].
Median municipal building index	Represents the proportion of sampled buildings infested with *Ae. aegypti* during the sampling period in each Brazilian municipality.	Used as an input for Boosted Regression Trees.	Collected by the Brazilian Ministry of Health using the LIRAa protocol [2–4]
Urban accessibility	Travel time (in minutes) to the nearest city with over 50,000 inhabitants.	Used as an input for Boosted Regression Trees.	Weiss et al. (2018) [5].
Population size	Projected municipal population sizes for 2024-2050.	Used as an input for Boosted Regression Trees.	Jones & O'Neill (2016) [6].
Urban land cover	Projects of mean municipal urban land cover for 2024-2050.	Used as an input for Boosted Regression Trees.	Zhou et al. (2019) [7].

References

1. O'Neill BC, Tebaldi C, van Vuuren DP, Eyring V, Friedlingstein P, Hurtt G, et al. The Scenario Model Intercomparison Project (ScenarioMIP) for CMIP6. Geosci Model Dev. 2016;9: 3461–3482. doi:10.5194/gmd-9-3461-2016

2. Secretaria de Vigilância Sanitária em Saúde. Diagnóstico rápido nos municípios para vigilância entomológica do *Aedes aegypti* no Brasil - LIRAa. Metodologia para avaliação dos índices de Breteau e predial. Brasília; 2005.

3. Coelho GE. Challenges in the control of *Aedes aegypti*. Rev Inst Med Trop Sao Paulo. 2012;54: 13–14.

4. Coelho RG, Lourenço-de-Oliveira R, Braga IA. Updating the geographical distribution and frequency of *Aedes albopictus* in Brazil with remarks regarding its range in the Americas. Mem Inst Oswaldo Cruz. 2014;109: 787–796.

5. Weiss DJ, Nelson A, Gibson HS, Temperley W, Peedell S, Lieber A, et al. A global map of travel time to cities to assess inequalities in accessibility in 2015. Nature. 2018;553: 333–336. doi:10.1038/nature25181

6. Jones B, O'Neill BC. Spatially explicit global population scenarios consistent with the Shared Socioeconomic Pathways. Environmental Research Letters. 2016;11: 084003. doi:10.1088/1748-9326/11/8/084003

7. Zhou Y, Varquez ACG, Kanda M. High-resolution global urban growth projection based on multiple applications of the SLEUTH urban growth model. Sci Data. 2019;6: 34. doi:10.1038/s41597-019-0048-z
