# Supplementary material for: Climate change, urbanisation and transmission potential: Aedes aegypti mosquito projections forecast future arboviral disease hotspots in Brazil
Source: PLoS Negl Trop Dis. 2025 Sep 18;19(9):e0013415. doi: 10.1371/journal.pntd.0013415 (PMC12445552; doi:10.1371/journal.pntd.0013415)
Supplement: S3 Table — (PDF) [file pntd.0013415.s011.pdf]

S3 Table. Parameter values and their source for calculation of R_0 for dengue fever.

Parameter	Description	Value	Source
a	*Ae. aegypti* biting rate	0.50	Reference [1]
B	Vector to human transmission efficiency	0.38	Reference [1]
C	Human to vector transmission efficiency	0.38	Reference [1]
r	Human recovery rate	0.17	Reference [1]

References

1. Alphey N, Alphey L, Bonsall MB. A model framework to estimate impact and cost of genetics-based sterile insect methods for dengue vector control. PLoS One. 2011;6: e25384.
