# Supplementary material for: Climate change, urbanisation and transmission potential: Aedes aegypti mosquito projections forecast future arboviral disease hotspots in Brazil
Source: PLoS Negl Trop Dis. 2025 Sep 18;19(9):e0013415. doi: 10.1371/journal.pntd.0013415 (PMC12445552; doi:10.1371/journal.pntd.0013415)
Supplement: S4 Table — (PDF) [file pntd.0013415.s012.pdf]

S4 Table. Model-estimated mean annual *Ae. aegypti* density (mosquitoes per km²) in Brazil's ten largest cities for 2024, 2030, 2050, and 2080 under four greenhouse gas emission scenarios: SSP1–2.6 (low), SSP2–4.5 and SSP3–7.0 (intermediate), and SSP5–8.5 (high). Within the table, cities are ordered geographically from north to south to reflect climatic gradients relevant to mosquito ecology.

City		SSP1-2.6			SSP2-4.5			SSP3-7.0			SSP5-8.5		
	2024	2030	2050	2080	2030	2050	2080	2030	2050	2080	2030	2050	2080
**Manaus**	1812	1845	1839	1866	1875	1890	1880	1890	1926	1855	1898	1891	1370
**Fortaleza**	1331	1354	1447	1497	1379	1518	1648	1396	1510	1858	1473	1594	1734
**Recife**	942	995	1068	1087	1016	1126	1256	1041	1150	1346	1004	1187	1293
**Salvador**	880	932	969	909	914	953	1140	885	1034	1070	901	1040	1164
**Brasília**	830	919	978	958	884	1014	1149	843	1054	1440	868	1100	1586
**Goiânia**	899	981	1046	1039	950	1069	1200	914	1112	1483	949	1170	1614
**Belo Horizonte**	540	633	652	672	578	663	770	567	726	1017	571	759	1208
**Rio de Janeiro**	638	703	737	772	666	748	847	658	762	998	646	848	1181
**São Paulo**	594	652	691	720	633	704	800	583	712	1003	589	778	1158
**Curitiba**	497	524	579	603	523	584	684	491	623	882	508	656	1003
