# Supplementary material for: Climate change, urbanisation and transmission potential: Aedes aegypti mosquito projections forecast future arboviral disease hotspots in Brazil
Source: PLoS Negl Trop Dis. 2025 Sep 18;19(9):e0013415. doi: 10.1371/journal.pntd.0013415 (PMC12445552; doi:10.1371/journal.pntd.0013415)
Supplement: S5 Table — (PDF) [file pntd.0013415.s013.pdf]

S5 Table. Model-estimated mean annual *Ae. aegypti* density (mosquitoes per km^2) in Brazil's five geographical regions for 2024, 2030, 2050, and 2080 under four greenhouse gas emission scenarios: SSP1–2.6 (low), SSP2–4.5 and SSP3–7.0 (intermediate), and SSP5–8.5 (high).

**Region**		**SSP1-2.6**			**SSP2-4.5**			**SSP3-7.0**			**SSP5-8.5**		
	**2024**	**2030**	**2050**	**2080**	**2030**	**2050**	**2080**	**2030**	**2050**	**2080**	**2030**	**2050**	**2080**
**Northeast**	1053	1116	1167	1181	1111	1242	1406	1101	1290	1551	1114	1322	1601
**North**	1151	1178	1231	1248	1202	1277	1322	1177	1298	1375	1214	1317	1265
**Southeast**	749	825	871	878	783	891	1008	762	930	1237	767	992	1434
**Central-West**	1126	1196	1244	1250	1180	1284	1368	1156	1297	1484	1171	1361	1499
**South**	564	580	656	683	592	679	786	576	726	974	598	766	1095
