# Supplementary material for: Climate change, urbanisation and transmission potential: Aedes aegypti mosquito projections forecast future arboviral disease hotspots in Brazil
Source: PLoS Negl Trop Dis. 2025 Sep 18;19(9):e0013415. doi: 10.1371/journal.pntd.0013415 (PMC12445552; doi:10.1371/journal.pntd.0013415)
Supplement: S6 Table — (PDF) [file pntd.0013415.s014.pdf]

S6 Table. Model-estimated mean annual *Ae. aegypti* density (mosquitoes per km²) in Brazil's 27 states for 2024, 2030, 2050, and 2080 under four greenhouse gas emission scenarios: SSP1–2.6 (low), SSP2–4.5 and SSP3–7.0 (intermediate), and SSP5–8.5 (high). Within the table, states are ordered geographically from north to south to reflect climatic gradients relevant to mosquito ecology.

State		SSP1-2.6			SSP2-4.5			SSP3-7.0			SSP5-8.5		
	**2024**	**2030**	**2050**	**2080**	**2030**	**2050**	**2080**	**2030**	**2050**	**2080**	**2030**	**2050**	**2080**
**Roraima**	1005	1007	1076	1068	1035	1105	1146	1024	1132	1152	1048	1108	1055
**Amapá**	861	898	918	944	887	923	980	903	966	1055	878	946	1031
**Amazonas**	1192	1212	1267	1280	1230	1301	1321	1203	1321	1342	1264	1344	1177
**Pará**	1092	1117	1158	1182	1155	1224	1264	1133	1232	1347	1146	1245	1253
**Maranhão**	1431	1480	1535	1594	1515	1674	1808	1491	1682	2044	1524	1728	1982
**Ceará**	1300	1355	1438	1491	1379	1551	1735	1386	1586	1997	1419	1639	1956
**Rio Granda do Norte**	1278	1339	1392	1438	1325	1500	1661	1384	1530	1839	1364	1554	1758
**Paraíba**	1029	1093	1142	1190	1093	1233	1404	1126	1282	1555	1097	1298	1563
**Piauí**	1129	1195	1253	1275	1196	1342	1525	1177	1394	1702	1197	1432	1745
**Pernambuco**	912	982	1028	1049	972	1097	1270	977	1147	1375	957	1177	1446
**Alagoas**	923	967	1023	1026	986	1083	1214	975	1102	1250	935	1146	1267
**Acre**	1210	1251	1325	1351	1273	1397	1472	1225	1419	1601	1311	1482	1583
**Rondônia**	1416	1459	1562	1569	1482	1578	1667	1456	1619	1703	1502	1675	1601
**Sergipe**	925	965	1016	989	976	1055	1217	933	1081	1180	923	1126	1215
**Tocantins**	1172	1244	1286	1313	1254	1360	1498	1212	1407	1601	1224	1428	1621
**Bahia**	757	830	867	835	791	888	1056	776	963	1119	787	982	1275
**Mato Grosso**	1180	1253	1295	1303	1244	1350	1442	1216	1367	1529	1245	1426	1528
**Goiás**	1032	1124	1183	1179	1088	1219	1347	1053	1248	1591	1083	1325	1703
**Distrito Federal**	842	934	993	972	897	1030	1168	855	1070	1463	881	1118	1616
**Minas Gerais**	691	781	817	817	729	837	962	713	889	1190	722	943	1398
**Espírito Santo**	785	864	871	894	797	904	998	790	970	1145	841	988	1397
**Mato Grosso do Sul**	1086	1127	1179	1190	1112	1185	1208	1111	1172	1274	1075	1238	1236
**Rio de Janeiro**	709	776	807	849	730	830	934	717	856	1118	723	933	1349
**São Paulo**	884	931	1007	1020	917	1024	1131	879	1032	1383	866	1119	1538
**Paraná**	754	785	861	895	786	886	1014	764	924	1242	762	996	1398
**Santa Catarina**	453	478	549	570	484	572	679	469	615	879	489	642	981
**Rio Grande do Sul**	467	471	549	572	492	569	661	480	625	816	520	646	920
