# Supplementary material for: Climate change, urbanisation and transmission potential: Aedes aegypti mosquito projections forecast future arboviral disease hotspots in Brazil
Source: PLoS Negl Trop Dis. 2025 Sep 18;19(9):e0013415. doi: 10.1371/journal.pntd.0013415 (PMC12445552; doi:10.1371/journal.pntd.0013415)
Supplement: S7 Table — (PDF) [file pntd.0013415.s015.pdf]

S7 Table. Model-estimated fold-change in mean annual *Ae. aegypti* density (mosquitoes per km²) from 2024 in Brazil's ten largest cities for 2030, 2050, and 2080 under four greenhouse gas emission scenarios: SSP1–2.6 (low), SSP2–4.5 and SSP3–7.0 (intermediate), and SSP5–8.5 (high). Within the table, cities are ordered geographically from north to south to reflect climatic gradients relevant to mosquito ecology.

City	SSP1-2.6			SSP2-4.5			SSP3-7.0			SSP5-8.5		
	2030	2050	2080	2030	2050	2080	2030	2050	2080	2030	2050	2080
**Manaus**	1.02	1.01	1.03	0.98	0.99	0.98	0.99	1.01	0.97	1.01	1.01	0.73
**Fortaleza**	1.02	1.09	1.12	1.03	1.13	1.23	1.04	1.13	1.38	1.09	1.18	1.29
**Recife**	1.06	1.13	1.15	1.04	1.15	1.29	1.07	1.18	1.38	1.06	1.25	1.36
**Salvador**	1.06	1.10	1.03	1.07	1.12	1.34	1.04	1.21	1.25	1.04	1.20	1.35
**Brasília**	1.11	1.18	1.15	1.06	1.21	1.38	1.01	1.26	1.72	1.06	1.34	1.93
**Goiânia**	1.09	1.16	1.16	1.05	1.18	1.33	1.01	1.23	1.64	1.08	1.33	1.83
**Belo Horizonte**	1.17	1.21	1.24	1.05	1.20	1.39	1.03	1.31	1.84	1.02	1.36	2.16
**Rio de Janeiro**	1.10	1.16	1.21	1.07	1.20	1.36	1.05	1.22	1.60	0.95	1.25	1.74
**São Paulo**	1.10	1.16	1.21	1.13	1.25	1.42	1.04	1.27	1.79	0.98	1.29	1.92
**Curitiba**	1.05	1.17	1.21	1.10	1.23	1.44	1.04	1.32	1.86	1.01	1.30	1.99
