# Supplementary material for: Climate change, urbanisation and transmission potential: Aedes aegypti mosquito projections forecast future arboviral disease hotspots in Brazil
Source: PLoS Negl Trop Dis. 2025 Sep 18;19(9):e0013415. doi: 10.1371/journal.pntd.0013415 (PMC12445552; doi:10.1371/journal.pntd.0013415)
Supplement: S8 Table — (PDF) [file pntd.0013415.s016.pdf]

S8 Table. Model-estimated fold-change in mean annual *Ae. aegypti* density (mosquitoes per km^2) from 2024 in Brazil's five geographical regions for 2030, 2050, and 2080 under four greenhouse gas emission scenarios: SSP1–2.6 (low), SSP2–4.5 and SSP3–7.0 (intermediate), and SSP5–8.5 (high).

**Region**	**SSP1-2.6**			**SSP2-4.5**			**SSP3-7.0**			**SSP5-8.5**		
	**2030**	**2050**	**2080**	**2030**	**2050**	**2080**	**2030**	**2050**	**2080**	**2030**	**2050**	**2080**
**Northeast**	1.06	1.11	1.12	1.05	1.17	1.32	1.04	1.21	1.46	1.05	1.25	1.51
**North**	1.02	1.07	1.08	1.03	1.09	1.13	1.01	1.11	1.18	1.04	1.13	1.09
**Southeast**	1.10	1.16	1.17	1.07	1.21	1.37	1.04	1.27	1.68	1.03	1.33	1.92
**Central-West**	1.06	1.10	1.11	1.04	1.14	1.21	1.02	1.15	1.31	1.03	1.20	1.32
**South**	1.03	1.16	1.21	1.08	1.24	1.44	1.05	1.33	1.78	1.04	1.34	1.89
