# Supplementary material for: Climate change, urbanisation and transmission potential: Aedes aegypti mosquito projections forecast future arboviral disease hotspots in Brazil
Source: PLoS Negl Trop Dis. 2025 Sep 18;19(9):e0013415. doi: 10.1371/journal.pntd.0013415 (PMC12445552; doi:10.1371/journal.pntd.0013415)
Supplement: S9 Table — (PDF) [file pntd.0013415.s017.pdf]

S9 Table. Model-estimated fold-change in mean annual *Ae. aegypti* density (mosquitoes per km^2) from 2024 in Brazil's 27 states for 2030, 2050, and 2080 under four greenhouse gas emission scenarios: SSP1–2.6 (low), SSP2–4.5 and SSP3–7.0 (intermediate), and SSP5–8.5 (high). Within the table, states are ordered geographically from north to south to reflect climatic gradients relevant to mosquito ecology.

State	SSP1-2.6			SSP2-4.5			SSP3-7.0			SSP5-8.5		
	**2030**	**2050**	**2080**	**2030**	**2050**	**2080**	**2030**	**2050**	**2080**	**2030**	**2050**	**2080**
**Roraima**	1.00	1.07	1.06	1.03	1.10	1.14	1.02	1.13	1.15	1.07	1.13	1.08
**Amapá**	1.04	1.07	1.10	0.99	1.03	1.09	1.01	1.08	1.18	1.04	1.13	1.23
**Amazonas**	1.02	1.06	1.07	1.02	1.08	1.10	1.00	1.10	1.11	1.04	1.11	0.97
**Pará**	1.02	1.06	1.08	1.03	1.09	1.12	1.01	1.09	1.20	1.04	1.13	1.14
**Maranhão**	1.03	1.07	1.11	1.03	1.14	1.23	1.01	1.14	1.39	1.04	1.18	1.35
**Ceará**	1.04	1.11	1.15	1.03	1.16	1.30	1.04	1.19	1.50	1.07	1.24	1.48
**Rio Granda do Norte**	1.05	1.09	1.13	1.02	1.16	1.28	1.07	1.18	1.42	1.08	1.23	1.39
**Paraíba**	1.06	1.11	1.16	1.04	1.17	1.34	1.07	1.22	1.48	1.08	1.27	1.53
**Piauí**	1.06	1.11	1.13	1.05	1.17	1.33	1.03	1.22	1.49	1.04	1.25	1.52
**Pernambuco**	1.08	1.13	1.15	1.05	1.19	1.37	1.06	1.24	1.49	1.06	1.30	1.60
**Alagoas**	1.05	1.11	1.11	1.07	1.17	1.31	1.05	1.19	1.35	1.04	1.27	1.40
**Acre**	1.03	1.10	1.12	1.06	1.17	1.23	1.02	1.19	1.34	1.03	1.17	1.25
**Rondônia**	1.03	1.10	1.11	1.04	1.11	1.17	1.02	1.14	1.20	1.04	1.16	1.11
**Sergipe**	1.04	1.10	1.07	1.07	1.16	1.34	1.03	1.19	1.30	1.03	1.26	1.36
**Tocantins**	1.06	1.10	1.12	1.05	1.14	1.25	1.01	1.17	1.34	1.04	1.21	1.37
**Bahia**	1.10	1.14	1.10	1.07	1.2	1.43	1.05	1.30	1.51	1.06	1.32	1.72
**Mato Grosso**	1.06	1.10	1.11	1.04	1.13	1.21	1.02	1.14	1.28	1.04	1.19	1.28
**Goiás**	1.09	1.15	1.14	1.06	1.18	1.31	1.02	1.21	1.55	1.06	1.30	1.67
**Distrito Federal**	1.11	1.18	1.15	1.06	1.22	1.38	1.01	1.26	1.73	1.06	1.34	1.94
**Minas Gerais**	1.13	1.18	1.18	1.06	1.21	1.39	1.03	1.29	1.72	1.05	1.37	2.03
**Espírito Santo**	1.10	1.11	1.14	1.03	1.17	1.29	1.02	1.26	1.48	1.09	1.29	1.82
**Mato Grosso do Sul**	1.04	1.09	1.10	1.04	1.11	1.13	1.04	1.09	1.19	0.99	1.14	1.14
**Rio de Janeiro**	1.09	1.14	1.20	1.07	1.21	1.37	1.05	1.25	1.64	0.99	1.28	1.84
**São Paulo**	1.05	1.14	1.15	1.09	1.22	1.35	1.05	1.23	1.65	0.99	1.28	1.76
**Paraná**	1.04	1.14	1.19	1.09	1.23	1.41	1.06	1.28	1.73	1.01	1.32	1.86
**Santa Catarina**	1.05	1.21	1.26	1.08	1.28	1.52	1.05	1.38	1.97	1.05	1.38	2.10
**Rio Grande do Sul**	1.01	1.18	1.23	1.07	1.24	1.44	1.05	1.36	1.78	1.08	1.34	1.91
