# Supplementary material for: Climate change, urbanisation and transmission potential: Aedes aegypti mosquito projections forecast future arboviral disease hotspots in Brazil
Source: PLoS Negl Trop Dis. 2025 Sep 18;19(9):e0013415. doi: 10.1371/journal.pntd.0013415 (PMC12445552; doi:10.1371/journal.pntd.0013415)
Supplement: S10 Table — (PDF) [file pntd.0013415.s018.pdf]

S10 Table. Details of temporal data of *Ae. aegypti* used to validate model predictions. The location, citation and trap type for each dataset are shown. The ${r_{xy}}^2$value is the correlation coefficient for the modified Chelton method. The p-value indicates whether model estimates were significantly correlated with the time series data. Whether or not studies provide the raw time series data within their supplementary information is indicated; where raw data were not available, data were digitised from figures using WebPlotDigitizer, as detailed in the main text [1]. The data source for each study is indicated. This specifies whether data taken from each study were generated by the study authors or obtained from a co-operative third party who have not made the data publicly available themselves. All published data were licensed under CC BY 4.0 (https://creativecommons.org/licenses/by/4.0/), permitting reuse of content with appropriate credit and sourcing of original authors.

Location	Study	Trap type	${r_{xy}}^2$ (95%CI)	P-value	Number of traps	Sampling frequency	Raw data available?	Data source
Manaus	Degener et al. (2014) [2]	BG-Sentinal	0.11 (0.00, 0.33)	0.17	24	14 days	N	Author collection
	Degener et al. (2014) [2]	MosquiTrap	0.08 (0.00, 0.29)	0.28	24	14 days	N	Author collection
Campo Grande	Codeço et al. (2015) [3]	BG-Sentinal	0.61 (0.22, 0.85)	0.03	24	30 days	N	Author collection
Parnamirim	Codeço et al. (2015) [3]	BG-Sentinal	0.10 (0.05, 0.49)	0.32	24	30 days	N	Author collection
Duque de Caxias	Codeço et al. (2015) [3]	BG-Sentinal	0.58 (0.11, 0.86)	0.04	24	30 days	N	Author collection
Novo Iguaçu	Codeço et al. (2015) [3]	BG-Sentinal	0.74 (0.32, 0.92)	0.04	24	30 days	N	Dengue-MI
Porto Alegre	da Cruz Ferreira et al. (2017) [4]	MosquiTrap	0.80 (0.66, 0.89)	0.003	712	7 days	N	Dengue-MI
Vitória	Pepin et al. (2015) [5]	MosquiTrap	0.32 (0.23, 0.41)	<0.001	1392	7 days	Y	Dengue-MI
Governador Valadares	Pepin et al. (2015) [5]	MosquiTrap	0.15 (0.06, 0.28)	0.002	373	7 days	Y	Dengue-MI
Sete Lagoas	Pepin et al. (2015) [5]	MosquiTrap	0.44 (0.32, 0.56)	<0.001	411	7 days	Y	Dengue-MI

References

1. Rohatgi A. WebPlotDigitizer. [cited 6 Feb 2025]. Available: https://automeris.io/
2. Degener CM, Ázara TMF de, Roque RA, Codeço CT, Nobre AA, Ohly JJ, et al. Temporal abundance of Aedes aegypti in Manaus, Brazil, measured by two trap types for adult mosquitoes. Mem Inst Oswaldo Cruz. 2014;109: 1030–40. doi:10.1590/0074-0276140234
3. Codeço CT, Lima AWS, Araújo SC, Lima JBP, Maciel-de-Freitas R, Honório NA, et al. Surveillance of Aedes aegypti: comparison of house index with four alternative traps. PLoS Negl Trop Dis. 2015;9: e0003475. doi:10.1371/journal.pntd.0003475
4. da Cruz Ferreira DA, Degener CM, de Almeida Marques-Toledo C, Bendati MM, Fetzer LO, Teixeira CP, et al. Meteorological variables and mosquito monitoring are good predictors for infestation trends of Aedes aegypti, the vector of dengue, chikungunya and Zika. Parasit Vectors. 2017;10: 78. doi:10.1186/s13071-017-2025-8
5. Pepin KM, Leach CB, Marques-Toledo C, Laass KH, Paixao KS, Luis AD, et al. Utility of mosquito surveillance data for spatial prioritization of vector control against dengue viruses in three Brazilian cities. Parasit Vectors. 2015;8: 98. doi:10.1186/s13071-015-0659-y
